# Mitochondrial Genomes Reveal Slow Rates of Molecular Evolution and the Timing of Speciation in Beavers (*Castor*), One of the Largest Rodent Species

**DOI:** 10.1371/journal.pone.0014622

**Published:** 2011-01-28

**Authors:** Susanne Horn, Walter Durka, Ronny Wolf, Aslak Ermala, Annegret Stubbe, Michael Stubbe, Michael Hofreiter

**Affiliations:** 1 Max Planck Institute for Evolutionary Anthropology, Leipzig, Germany; 2 Department for Community Ecology, Helmholtz-Centre for Environmental Research-UFZ, Halle, Germany; 3 Department for Molecular Evolution and Animal Systematics, University of Leipzig, Leipzig, Germany; 4 Finnish Game and Fisheries Research Institute, Helsinki, Finland; 5 Institute of Zoology, University of Halle-Wittenberg, Wittenberg, Germany; Texas A&M University, United States of America

## Abstract

**Background:**

Beavers are one of the largest and ecologically most distinct rodent species. Little is known about their evolution and even their closest phylogenetic relatives have not yet been identified with certainty. Similarly, little is known about the timing of divergence events within the genus *Castor*.

**Methodology/Principal Findings:**

We sequenced complete mitochondrial genomes from both extant beaver species and used these sequences to place beavers in the phylogenetic tree of rodents and date their divergence from other rodents as well as the divergence events within the genus *Castor*. Our analyses support the phylogenetic position of beavers as a sister lineage to the scaly tailed squirrel *Anomalurus* within the mouse related clade. Molecular dating places the divergence time of the lineages leading to beavers and *Anomalurus* as early as around 54 million years ago (mya). The living beaver species, *Castor canadensis* from North America and *Castor fiber* from Eurasia, although similar in appearance, appear to have diverged from a common ancestor more than seven mya. This result is consistent with the hypothesis that a migration of *Castor* from Eurasia to North America as early as 7.5 mya could have initiated their speciation. We date the common ancestor of the extant Eurasian beaver relict populations to around 210,000 years ago, much earlier than previously thought. Finally, the substitution rate of *Castor* mitochondrial DNA is considerably lower than that of other rodents. We found evidence that this is correlated with the longer life span of beavers compared to other rodents.

**Conclusions/Significance:**

A phylogenetic analysis of mitochondrial genome sequences suggests a sister-group relationship between *Castor* and *Anomalurus*, and allows molecular dating of species divergence in congruence with paleontological data. The implementation of a relaxed molecular clock enabled us to estimate mitochondrial substitution rates and to evaluate the effect of life history traits on it.

## Introduction

Dating back to approximately 40 million years, today the family of beavers, Castoridae, is represented by only two extant species, *Castor canadensis* in North America and *Castor fiber* in Eurasia. Both species are characterized by their large body size, being the second largest rodent, and their semi-aquatic lifestyle [1], [2]. However, several aspects of the early history and evolution of beavers remain unclear. Earlier attempts of their phylogenetic placement relative to other rodents were difficult because of a lack of fixed morphological differences, poor taxon sampling in many genetic studies, limited sequence data in previous studies, and contemporaneous radiations of multiple rodent lineages. More recent molecular data strongly support the placement of *Castor* within a “mouse-related clade,” containing several families including Pedetidae, Anomaluridae, Muridae, Dipodidae, Geomyidae, and Heteromyidae [3], [4], [5], [6]. Previous multigene studies have suggested Geomyoidea to be the closest relatives of beavers [5], [6]. However, the branches leading to both groups diverged very early in rodent evolution and transposon insertion analyses are inconclusive with regard to their monophyly [7], [8].

**Figure 1:**
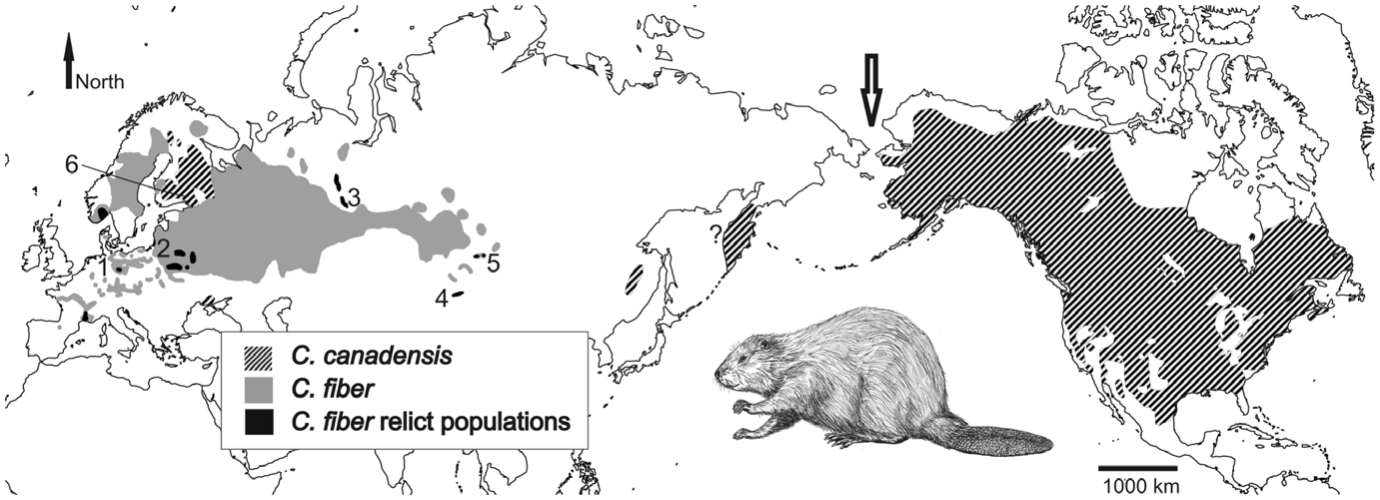
Distribution of beavers and sampling sites. Beavers live in North America (*C. canadensis*, striped areas) and Eurasia (*C. fiber*, grey areas). Black areas mark *C. fiber* relict populations from which the current populations developed. *C. canadensis* was introduced in Europe and Asia (striped areas, distribution not exactly known for Kamchatka). Numbers indicate beaver populations sampled for mitochondrial genome sequencing. *C. fiber* was sampled in the areas of the relict populations (1,3,4,5) or close to them (2) [31]. *C. canadensis* was sampled in a European introduced population (6). Arrow: migration via the Bering land bridge is suggested to have initiated *Castor* speciation around 8–7.6 million years ago (mya). Map redrawn from [1], [31] and [55].

Not only the phylogenetic placement of beavers within rodents is not completely understood, little is also known about the timing of the speciation event leading to the two extant beaver species. Both have a strikingly similar phenotype, making them almost indistinguishable in the field; therefore molecular methods are applied to differentiate them [1], [9]. Despite the morphological similarities, subtle morphological and biochemical features and different chromosome numbers support their distinction as different species [10]. The fossil record provides further information on the timing of *Castor* speciation. The appearance of the genus *Castor* in Eurasia and North America was estimated to the late Miocene and the Pliocene, between 9–4.9 mya [11], [12], [13], [14], [15], [16]. *Castor* is assumed to have emerged in Eurasia as a close relative of *Steneofiber* [11], [12], [14], [15], [16], and to have subsequently dispersed to North America via the Bering landbridge [12], [13], [15], [16], [17]. This dispersal event was estimated to 4.9–6.6 mya [13], [15]. However, since the estimates for the earliest appearance of *Castor* on both continents overlap, it is not entirely clear in which direction the dispersal of *Castor* took place. Irrespective of dispersal direction, since the two extant species of *Castor* are native to either Eurasia or North America, it seems reasonable to assume that a migration across the Bering strait could have initiated the speciation of *C. canadensis* and *C. fiber* [10], [14], [15] (Fig. 1). However, no study to date attempted obtaining a sequence based molecular date for the timing of divergence between the two species. As mitochondrial genomes are available for a number of rodent taxa [18], the addition of *Castor* mitochondrial genomes could shed light not only on the deep phylogeny of beavers, but also on the timing of divergence to other rodents and within the genus itself. Finally, mitochondrial genome data should allow for determination of the overall substitution rate in beaver mitochondrial DNA, which allows retrospectively investigating and dating population genetic processes.

Here we present the first complete mitochondrial genomes of beavers. We use these DNA sequences to investigate the phylogenetic position of beavers within rodents, date evolutionary events within the extant members of the family Castoridae and explore the substitution rate of their mitochondrial DNA.

## Results

### Phylogenetics

**Figure 2:**
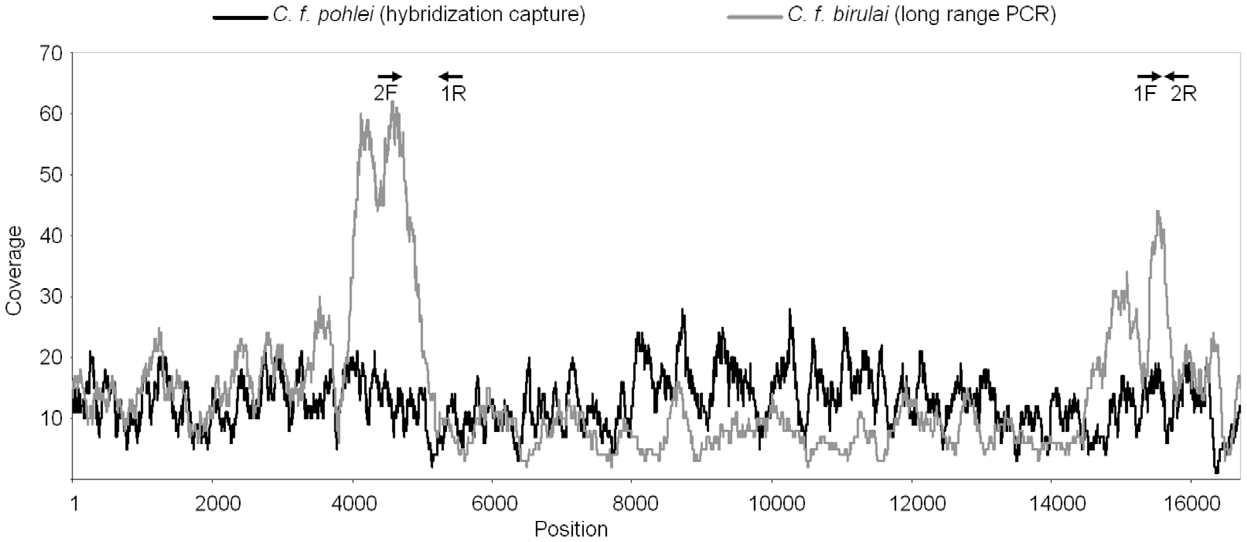
Sequencing coverage plots of beaver mitochondrial genomes. Plots for *C.f.* ssp. *birulai* amplified by long range PCR (grey line) and *C.f.* ssp. *pohlei* enriched by hybridization capture (black line). Coverage was more even when hybridization capture was used instead of long range PCR. Peaks of sequencing coverage are visible for *C.f.* ssp. *birulai* (grey) in proximity to priming sites (indicated on top of the plots).

**Figure 3:**
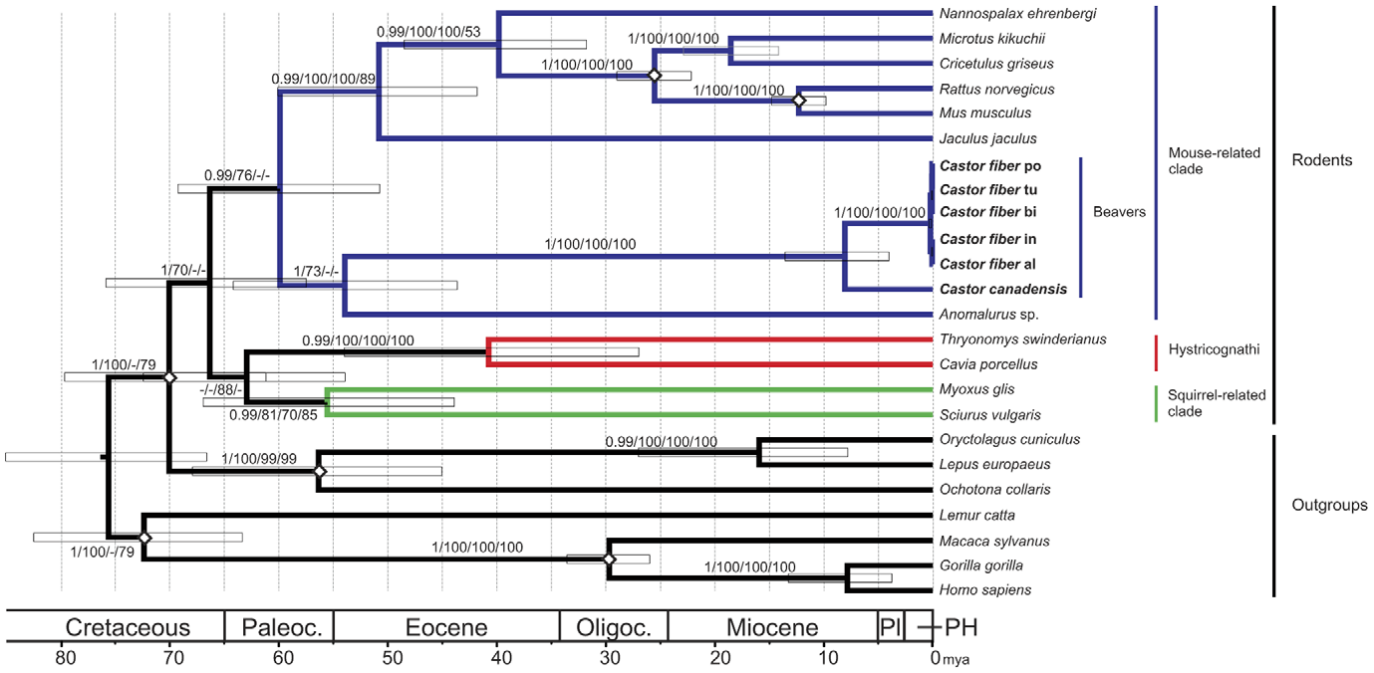
Timetree of rodents and outgroups. Beavers share a common ancestor with Anomaluromorpha around 54 mya (CI: 44–64 mya); the only extant beaver genus *Castor* separated into two species from 8–7.6 mya onwards (CI: 3.7–13 mya). The common ancestor of the Eurasian relict populations was estimated to have lived around 210,000 ya (CI: 0.11–0.34 mya). The tree depicted is a maximum clade credibility tree from BEAST analyses based on a 16,352 bp alignment (including gaps). Bayesian posterior probability (>0.6) and bootstrap support values (>50) are shown at the branches and separated by slashes for MrBayes, maximum likelihood, neighbor joining and maximum parsimony, respectively. Diamonds indicate fossil calibrations (Table S4). Paleoc.: Paleocene; Oligoc.: Oligocene; Pl: Pliocene, PH: Pleistocene and Holocene. *Castor fiber* po, tu, bi, in and al indicate the subspecies sampled (see Table S1).

We sequenced mitochondrial genomes of one *C. canadensis* and five *C. fiber* utilizing long range PCR, DNA hybridization capture and 454 sequencing (Table S1 and and Supporting References S1). Assemblies derived from hybridization capture showed a considerably smoother read distribution than those derived from long range PCR (Fig. 2). The consensus sequences obtained were aligned with a variety of rodent and outgroup mitochondrial genomes for phylogenetic analyses and molecular dating (Table S2). We employed several different methods for investigating the phylogenetic relationships in our dataset. All methods, Bayesian approaches, maximum likelihood (ML), neighbor joining (NJ) and maximum parsimony (MP) recovered a well supported, monophyletic clade of the *Castor* individuals. In Fig. 3 we present a maximum clade credibility tree from BEAST with support values from all phylogenetic methods. Highest support was obtained for the sister group relationship of *C. canadensis* and *C. fiber*, the latter represented by a monophyletic clade of five subspecies (*C. fiber* ssp. *albicus*, *belorussicus/orientoeuropaeus*, *birulai*, *tuvinicus*, and *pohlei*). A position of *Castor* within the mouse related clade of rodents, as sister to the scaly tailed squirrel *Anomalurus*, was supported with very high and maximum support, respectively, from Bayesian analyses as well as with bootstrap values higher than 70 in ML analyses. The squirrel related clade and Hystricognathi were recovered with high support values in all phylogenetic methods. Although the monophyly of rodents was not supported in NJ and MP, it received maximum support from Bayesian and a bootstrap value of 70 in ML analyses. However, the relationship between the three major rodent clades remains enigmatic, since their branching order could not be resolved. Since difficulties in resolving the root of the rodent tree have been attributed to the effect of long branch attraction, causing rodent sequences to cluster with outgroups [18], [19], we conducted additional ML analyses with a more representative sample of outgroup sequences (Table S2). These additional outgroup sequences could not improve the resolution of the early branchings, instead, the ML bootstrap support value for rodent monophyly decreased from 70 to 52 (Table S3). However, since the monophyly of rodents was supported in our Bayesian and ML trees based on the smaller set of taxa as well as other studies on nuclear DNA sequences and transposon insertions [7], [8], we nevertheless attempted estimating the time elapsed since a monophyletic origin of all rodents using a molecular dating approach implemented in BEAST [20].

### Divergence estimates

We estimated that all rodents shared a common ancestor around 67 mya (CI: 57–76 mya) (Table S4). Around 54 mya (CI: 44–64 mya), the branch leading to beavers diverged from the common ancestor with the scaly tailed squirrel *Anomalurus*. The mitochondrial DNA of the two beaver species *C. fiber* and *C. canadensis* shared a most recent common ancestor between 8 mya (mean) and 7.6 mya (median) (CI: 3.7–13 mya; Table S4). Within Eurasian beavers, the individuals coming from different relict populations shared a common ancestor around 210,000 years ago (CI: 110,000-340,000 ya).

### Substitution rates

**Figure 4:**
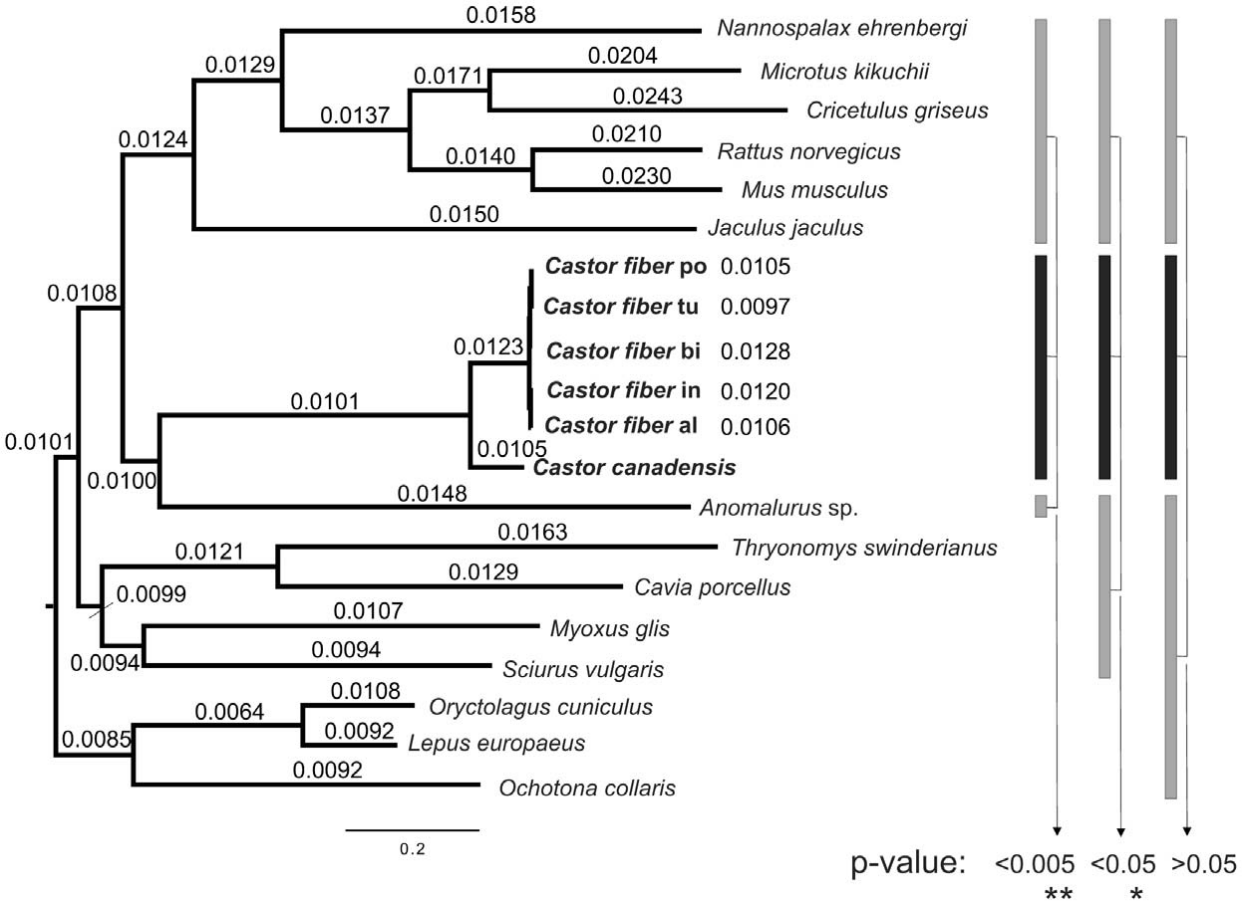
Comparison of mitochondrial substitution rates among glires. Phylogenetic tree of glires (rodents and lagomorphs) with branch lengths from the BEAST analysis. Mitochondrial substitution rates are shown on the branches or next to each taxon in units of substitutions per million years. Beavers exhibit short branches and substitution rates for beavers are the lowest within the mouse-related clade, significantly lower than those of most other rodents (see also Table 1).

As beavers are the largest rodents for which a mitochondrial genome sequence is currently available, we explored the mitochondrial substitution rate in comparison to other rodents. There is some evidence that the substitution rate is linked to life expectancy and body mass [21] even though this connection is controversial [22]. Animals like the beaver, with higher life expectancy and larger body mass are therefore expected to show decreased substitution rates. Molecular substitution rates for the taxa in our dataset were estimated in the BEAST analysis and are depicted on each branch in Fig. 4 (see also Table S5). The only rodents with a lower mitochondrial rate were *Sciurus* and *Myoxus* in the squirrel-related clade. Within the mouse-related clade the mitochondrial tip rates of beavers were significantly lower than those of the other members of this clade (p-value  = 0.005). Also, when the distribution of rates for beavers was compared to the distribution of rates for all other rodents in the dataset, the rates of beavers were significantly lower (p-value  = 0.014, Mann-Whitney U-test, Fig.4, Table 1). This difference became even more evident when instead of tip rates average rates leading to the individual taxa were used (see methods and Table S1). If tested across all glires (rodents and lagomorphs), the tip and average rates of beavers did not differ significantly anymore (Table 1).

**Table 1:** Mitochondrial substitution rates of *Castor* differ significantly from those of other rodents.

	Median rate at tips	Median rates averaged
*Castor* vs. rest of mouse-clade	U = 36, p-value = 0.004998 **	U = 42, p-value = 0.002591 **
*Castor* vs. rest of rodents	U = 58, p-value = 0.01375 *	U = 60, p-value = 0.007005 *
*Castor* vs. rest of glires	U = 62, p-value = 0.1075	U = 60, p-value = 0.1457

Results of Mann-Whitney U-tests testing the null hypothesis that substitution rates for *Castor* are identical to the rates of other rodents (Wilcoxon rank sum test with continuity correction as implemented in R).

*: significant, <0.05;

**: highly significant, <0.005.

**Figure 5:**
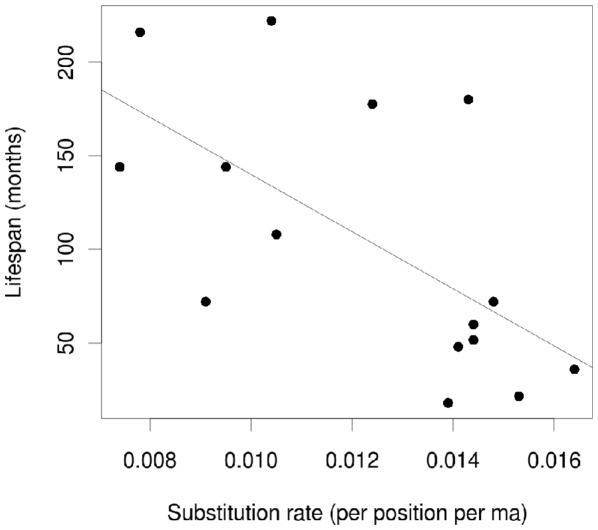
Linear regression of average substitution rates and lifespan of rodents. Rodents with higher mitochondrial substitution rates exhibit a shorter lifespan (R squared  = 0.4011, p-value  = 0.0113). See Table S6 and S7 for details.

We related the inferred substitution rates to body mass and life history traits of rodents as had been done earlier [21] (Fig. 5, Table S6). In linear regressions, more than 30 percent of the variation in tip rates (R^2^ = 0.34, p-value = 0.0225) and average rates (R^2^ = 0.40, p-value = 0.0113) could be explained by maximum lifespan. Thus considerably more variation in lifespan was attributable to mitochondrial rate variation than had been estimated previously (below 20 percent in [21]). However, for body mass and age at sexual maturity, no significant correlations with substitution rates could be identified (p-values>0.05, Table S6).

## Discussion

The phylogenetic relationships of extant rodent families have been difficult to resolve. However, several previous molecular studies recognized three major phylogenetic clades of rodents: the mouse-related clade, Ctenohystrica (relatives of the guinea pig) and the squirrel-related clade (Fig. 3). This branching was supported by nuclear DNA analyses [3], [5], [7], [8] as well as some studies including mitochondrial DNA [6], [23]. We find this phylogeny to be also supported by complete mitochondrial nucleotide sequences. In accordance with studies on nuclear DNA [3], [5], [6], *Anomalurus* is recovered as an early member of the mouse-related clade. In contrast, an earlier analysis of complete mitochondrial genome sequences did not recover *Anomalurus* within the mouse-related clade [18], possibly due to reducing the sequences to the coding regions for some of the phylogenetic inferences.

The root of the rodent tree is much more difficult to resolve. Although we obtain high support for rodents as a monophyletic group, confirming earlier studies [3], [5], [8], [24], [25], as in previous studies, we could not determine the branching order of the three major clades [3], [5], [18]. However, our analyses are based on mitochondrial DNA and therefore represent a single genomic locus only. Also, since many rodents exhibit high substitution rates, part of the lower resolution for the deeper nodes might result from mutational saturation of the sequences. Analyses of a larger number of nuclear loci should improve the resolution of the deeper phylogenetic nodes within rodents in the future.

We recovered the six beaver mitochondrial genomes in one monophyletic group within the mouse-related clade of rodents (Fig. 3). This phylogenetic position of beavers was indicated already in earlier analyses based on nuclear gene sequences and transposon insertions [5], [7], [8]. Now, mitochondrial genomics adds another line of evidence to this relationship. In our dataset, the mitochondrial sequence of the scaly tailed squirrel *Anomalurus* was most closely related to those of the beavers. However, there is no mitochondrial genome sequence available for the Geomyoidea (gophers and kangaroo rats), which have been found to be most closely related to beavers in multigene studies [5], [6] and could attach to the evolutionary branch leading to beavers even later than *Anomalurus*.

We estimated divergence times within rodents in BEAST using six fossil calibration points and a Bayesian relaxed molecular clock approach [20] and found all rodents to share a common ancestor around 67 mya (CI: 57–76 mya) (Table S4). This timing is remarkably coincident with the mass extinction event that marked the transition between the Cretaceous and Tertiary (K-T boundary). At that time dinosaurs went extinct and a major faunal change took place all over the world. The origin of rodents has been dated both earlier and later in other studies: around 96 mya [26], 71–89 mya [6], around 72 mya [3] and around 62 mya [18]. Our estimate lies in between the range of earlier estimates, close to the K-T boundary and predating the explosive radiation of rodents in the early Eocene (dated to around 55mya) [27]. Thus, the changing biota at the K-T boundary may have been causal for a first rodent radiation.

Around 54 mya (CI: 44–64 mya), a phylogenetic lineage leading to beavers diverged from its common ancestor with Anomaluromorpha (*Anomalurus* and *Pedetes*). Thus, beavers probably have a very long evolutionary history, which might explain their ecological and morphological peculiarities. In a previous study using 5.5 kb of nuclear and mitochondrial DNA sequences [3], the divergence of beavers and Anomaluromorpha was estimated even older, at more than 65 mya. However, since Geomyoidea are potentially even more closely related to beavers, additional DNA sequences for Geomyoidea will be required to provide information on the beginning of beaver evolution.

Similar to the divergence of the family Castoridae, the divergence time of the two extant beaver species has not yet been estimated with much precision. The origin of the extant beaver genus *Castor* has been suggested to lie in Eurasia at some time between 9.7 and 5.2 mya based on the fossil record and similarities with *Steneofiber* [14], [15], [16]. However, since there is overlap with the earliest appearance of *Castor* in North America (6.6 mya to 7.5 mya [12], [28]), the geographical origin of *Castor* remains uncertain. Independent of that, a migration of ancient *Castor* via Beringia and the subsequent geographical isolation of the two populations in Eurasia and North America, respectively, most likely led to the divergence of *Castor* lineages that ultimately gave rise to the modern species *C. fiber* and *C. canadensis*. According to our molecular datings, the two beaver species shared a common ancestor around 7.6–8 (CI: 3.7–13) mya. This molecular estimate is intriguingly close to the earliest fossil beaver remains in North America possibly dating as early as 7.5 mya [12]. The Beringian land bridge allowed faunal exchange between Eurasia and North America until its first flooding 5.4–5.5 mya and then again several times during the Pliocene and Pleistocene [29]. Thus, a migration over the Beringian landbridge around 8–7.6 mya has likely been the starting point for the speciation between *C. fiber* and *C. canadensis*, each on a different continent (Fig. 1).

Despite a successful evolutionary history across the Palaearctic for several millions of years, beaver populations decreased dramatically in size in more recent history. Extensive hunting by humans and habitat destruction affected both beaver species [1], [30] and left Eurasia with a few, isolated relict populations of *C. fiber* at the end of the 19^th^ century [30]. The diversification of these extant beaver populations was proposed to have happened during the last glacial period, from 115,000 ya onwards [31]. We dated the last common ancestor of the extant Eurasian beaver mitochondrial genomes to almost twice the age than estimated before, around 210,000 ya (CI: 110,000–340,00 ya). However, it has been pointed out that due to the problems in determining substitution rates precisely, such dates should be viewed with caution and correlating molecular divergence dates with changes in environmental conditions may be tentative at best [32]. It should be noted that when using fossil calibration points like in this study, the divergences towards the tips of the tree may be overestimated [33]. However, the substitution rate estimates for the tip branches leading to the individual subspecies are very similar to the overall estimate for the branch from the MRCA of all rodents to the tips of the beaver branches, suggesting that for this evolutionary lineage, time-dependent variation in substitution rates is negligible. Therefore, the diversification of the extant Eurasian beaver lineages started most likely substantially earlier than previously estimated. Also, geographically distinct lineages of other Eurasian, potentially forest dependant species showed similar divergence estimates, such as cave bears (173,000–414,000 ya [34]) and brown bears (174,000–314,000 ya [35]). The genetic diversity of these populations was probably shaped by climatic fluctuations during the glacial cycles since around 0.9 mya [36].

The analysis of substitution rates also shows that beavers display a lower substitution rate than most other rodents, a feature clearly visible in the branch length of the phylogenetic tree (Fig. 4). This difference was highly significant when beavers were tested against other members of the mouse-related clade. The rate difference was still significant when *Castor* was compared with all other rodents in the dataset, including the squirrel-related clade which also showed lower substitution rates. It has been argued before that certain morphological, physiological, and life history traits influence DNA substitution rates. For example, Welch et al. [21] showed that life history traits like body mass, lifespan and age at sexual maturity are negatively correlated with substitution rates over a wide range of mammals. Beavers are atypical among rodents with respect to several life history traits as they have a much larger body mass, longer lifespan and do not reach sexual maturity until 1.5–3 years of age (Table S7, [1], [14]). Thus, a lower substitution rate can be expected for beavers compared to other rodents. In our analysis of mitochondrial genomes, body mass and age at sexual maturity did not correlate significantly with mitochondrial substitution rates, although body mass did so earlier [21]. However, we found a significant negative correlation between the substitution rate and maximum lifespan as has been previously reported for mitochondrial synonymous sites across mammals [21]. Despite the smaller sample size in our analysis, a larger fraction of the variation in substitution rate was explained by maximum lifespan, even though we included non-synonymous sites, which did not show a significant correlation earlier [21]. Correlates of substitution rates with lifespan are not fully explained to date [21], but natural selection could have acted to reduce the mutation rate in mitochondria of long lived taxa such as the beaver [37], [38]. However, in a genome scan across 25 species, genes involved in DNA replication, repair or antioxidation did not show signatures of selection in long-lived taxa. Instead, the selected features were connected to cellular membrane and extracellular collagen composition and their functional relevance remains puzzling [39].

In summary, our study showed that over the wide taxonomic range of glires, datasets comprising whole mitochondrial genome sequences facilitate the inference of substitution rates, phylogenetic analyses and divergence estimates.

## Materials and Methods

### Ethics statement

The tissue sample of *Castor canadensis* was taken from a dead beaver, which was shot during the open hunting season in Finland. The sample of *Castor fiber* ssp. *albicus* came from a road kill, dissected with the approval from the environmental agency of Germany, administrative district of Leipzig. Samples *Castor fiber* ssp. *belorussicus*/*orientoeuropaeus*, *birulai*, *tuvinicus* and *pohlei* consisted of beaver tail skin or hair that was obtained from live animals captured with nets, live traps, and at night with a search light and netting from a boat. Animals were released afterward. Sampling procedures were consistent with guidelines of the American Society of Mammalogists for the capture and handling of mammals [40]. All catches were performed in cooperation with and under approval by, the Nature Reserve ‘Malaya Sosva’ in Russia, the Zhitkov Russian Research Institute of Game Management and Fur Farming in Kirov, Russia, as well as the National University of Mongolia in Ulaan-Baatar, Mongolia.

### Samples, DNA extraction and long range PCR

DNA was extracted from five tissue samples of *C. fiber* and from one sample of *C. canadensis* (Table S1) using the DNeasy blood & tissue kit (Quiagen). For well preserved DNA, the mitochondrial genome was amplified in two overlapping pieces around 11 and 6 kb in length, by long range PCR using the expand dNTPack (Roche) according to the manufacturer's instructions. Primer sequences are listed in Table S8. Long range PCR products were sheared using a Bioruptor UCD-200 (Diagenode). Barcoding adaptors with sample specific sequences and 454 sequencing adaptors were ligated to the sonicated products as described previously [41].

### DNA hybridization capture and sequencing

For less well preserved samples, for which long range PCR did not work, DNA hybridization capture was used to enrich for mitochondrial genomes (Table S1). For this process, biotinylated bait molecules are hybridized with a genomic library and later selectively captured on streptavidin beads [42]. Barcoded genomic libraries were prepared from *Castor* DNA as described previously [41], [43], allowing the simultaneous sequencing of different samples on the same 454 lane. To produce bait molecules, long range amplicons of *C. fiber* ssp. *albicus* were used. The PCR products were sheared until they had a length of around 300 bp, and double stranded, biotinylated adapters were ligated onto the ends. The hybridization mixture was set up as follows: 1 µg of genomic library, 100 ng of bait and four blocking oligos (each 2 µM) in 1x blocking Agent (Agilent) and 1x hybridization buffer (Agilent). Agilent reagents were from the aCGH Kit #5188-5220. Sequences for the blocking oligos are listed in Table S8. After denaturation of the mixture at 95°C for 5 min, the hybridization was carried out in 200 µl tubes (Eppendorf), rotating at 65°C for 48 hours in a conventional hybridization oven (SciGene).

After hybridization, biotinylated bait molecules were captured by incubation with 5 µl of magnetic streptavidin covered beads (Dynalbeads M270, Invitrogen) for 20 min at room temperature. The mixture was then placed into a magnetic rack (Beckman Coulter #A32782) allowing the separation of magnetic beads from the supernatant. The supernatant, containing non-target molecules was discarded and the remaining beads were washed five times using 1xBWT buffer (1 M NaCl, 10 mM Tris-Cl, 1 mM EDTA, 0.05% Tween-20 (Sigma), pH 8.0) and once in pre-warmed HW buffer (200 µl 10x AmpliTaq Gold buffer, 200 µl MgCl_2_, 1.6 ml H_2_O) at 50°C for 2 min. After one more wash with 1xBWT, the beads were transferred into a new tube with 100 µl of TE-buffer (containing 0.05% Tween-20). Finally, hybridized target molecules were separated from the bait molecules in 30 ul 1xTE by 5 min incubation at 95°C in a thermocycler. The eluate containing the sequencing library enriched for mitochondrial DNA, was directly used for quantification and sequencing.

### Sequence analyses and assembly

After 454 library preparation and quantification of the libraries by qPCR using emPCR priming sites [44], sequencing was carried out on the 454 FLX platform. *De novo* assembly of 454 reads of the mitochondrial genomes for *C. fiber* ssp. *albicus* and *C. canadensis* was done with runAssembly (454 Roche), separately for each of the two overlapping long range amplicons, and the overlaps joined by hand in BioEdit [45]. For the remaining *C. fiber* specimens, sequencing reads were mapped onto the assembled *C. fiber* mitochondrial genome using runMapping (454 software) and the iterative mapping tool IMA [46]. The output of the mappings was viewed with clview (software available at http://compbio.dfci.harvard.edu/tgi/software/) and the map aligner [46]. Mapping of the sequencing reads from one PCR derived sequence showed a somewhat uneven coverage and peaks of read counts in the proximity to priming sites (Fig. 2), which could result from amplicons that were aborted during the PCR shortly after priming. The generally observed differences in coverage between both long range PCR products might reflect variation in quantification or pooling of these prior to sequencing. Coverage plots from hybridization capture were smoother than those derived from long range PCR (Fig. 2). The obtained mappings covered the complete mitochondrial genomes for all samples. One site each in beaver samples *C. canadensis* and *C. fiber albicus* had less than 3x coverage, for which the sequence was confirmed by PCR and Sanger sequencing (sample specific primers listed in Table S8). Nuclear mitochondrial insertions (numts) could be ruled out for long range PCR by joining the overlaps creating a circular sequence, and are not expected to be a problem for hybridization capture since nuclear DNA has a much lower copy number than mitochondrial DNA. The length of the repeat region between the control region and tRNA-Phe is a minimum estimate, since the 454 sequencing reads did not span the region completely. For between two and 21 positions per mitochondrial genome, corrections of the assembled DNA sequence were made by hand in BioEdit. These affected mainly protein coding regions, where the length of homopolymers was corrected so that the reading frame for amino acid sequences was retained. The obtained sequences were deposited in GenBank with accession numbers FR691684-FR691689.

### Phylogenetic analyses and divergence estimates

Alignments of DNA sequences were done using mafft v6.708b [47] for the taxa listed in Table S2. The complete alignment including gaps had 19,419 bp in length. 3067 bp of the alignment containing the control region between tRNA-Pro and tRNA-Phe were not well aligned due to high sequence divergence and were therefore removed, resulting in an alignment of 16,352 bp for phylogenetic analyses.

Modeltest was used to determine the optimal evolutionary model for the dataset [48]. The general time reversible (GTR) model with a proportion of invariant sites of 0.2559 and a gamma shape parameter of 0.4721 was determined to be most appropriate. Using this substitution model, phylogenetic trees were calculated with maximum likelihood (ML). One hundred bootstrap replicates were done in Paup [49] version 4.0d105. The heuristic search used tree bisection reconnection (TBR) limited to 1000 rearrangements per bootstrap replicate due to computational constraints.

In order to test if the removal of fast evolving sites from the sequences could improve phylogenetic inference [6], two shorter versions of the original alignment were created and also subjected to ML inference (Table S3). One alignment contained all annotated loci with 15,865 bp (coding genes, l-rRNA, s-rRNA, replication origin, tRNAs) and thus excluded only 487 bp of non-annotated loci. The other alignment (containing coding genes, l-rRNA and s-rRNA) had 14,270 bp, excluding all tRNAs and the replication origin. Since these shortened alignments did not improve phylogenetic inference with ML (Table S3) and the longer alignment with 16,352 bp allowed the recognition of all major rodent clades, the latter was used for the following phylogenetic inferences and molecular dating with BEAST. The initial ML analyses on three alignments of different length were performed also on a larger taxon set in order to evaluate the topology of our phylogenetic tree, especially the monophyly of rodents. This larger taxon set comprised all 39 taxa listed in Table S2. Due to computational constraints, the taxon set was then reduced to 24 taxa, excluding some of the outgroup sequences for the following phylogenetic inferences and molecular dating with BEAST.

For neighbor joining (NJ) the T3P model was used in Mega4 [50]. 1,000 Bootstrap replicates were calculated with pairwise deletion and gamma distributed rates among sites (gamma shape parameter was 0.4729). For maximum parsimony (MP) 1,000 bootstrap replicates were done with close neighbor interchange (CNI, level 1) and initial trees for the CNI search by random addition trees (10 replications) in Mega4. Trees were searched in MrBayes v.3.1.2 with four parallel search chains for 10 million generations in triplicates. The search chains reached similar posterior probability levels in the triplicates as viewed in Tracer v1.4.1, final ESS were 339. The treefiles were combined in logcombiner and maximum clade credibility trees were created with TreeAnnotator implemented in BEAST v.1.5.3 and viewed in FigTree v.1.3.1 [51].

Molecular dating was carried out with BEAST v.1.5.3, a coalescence based method for parameter estimation [52]. Six fossil calibrations were taken into account as prior assumptions on divergence times (diamonds in Fig. 3, Table S4) and simultaneously optimized with other parameters describing the phylogeny. The relaxed molecular clock models implemented in BEAST assume independent rates on different branches of a phylogeny, thus allowing for calibrations in rather distantly related taxonomic groups. Thus, BEAST is specifically suited to accommodate the rate variation between lagomorphs, rodents and primates as well as that between rodents with slower and faster rates. The sampling priors for fossil calibrations were set to be normally distributed, incorporating knowledge on divergence estimates, such as the earliest appearance of members of a clade in the fossil record as well as the age of an assumed monophyletic origin and early relatives of the clade. Since there are uncertainties for determining the age of fossils and times that lineages actually diverged, we used rather wide ranges around these events for calibration (Table S4). Further, we used estimated base frequencies, an uncorrelated lognormal relaxed molecular clock, the Yule prior for speciation scenarios and UPGMA starting trees. BEAST xml-files were created in beauty (implemented in BEAST). Search chains were run in BEAST for 50 million generations and log files were written every 5,000 generations. The GTR substitution model was used and the first 10 percent of runs were discarded as burnin. Logcombiner from the BEAST package did not remove the burnin correctly, when combining logfiles. Therefore, logfiles of triplicates of the 50 million generation runs were combined into a single logfile by hand and the results were viewed in Tracer version v1.4.1 [53]. The posterior distributions for the fossil calibrations were visually inspected in Tracer v1.4.1 and verified to be symmetrically bell shaped, indicating a proper sampling from the prior distribution. A maximum clade credibility tree was created with treeannotator and viewed in FigTree.

### Comparison of evolutionary rates

Substitution rates for the branches leading to different rodent taxa were determined in the BEAST analyses (combined from three 50 million generation runs) and shown as branch labels in Figtree. We tested if beavers differ in substitution rates from other rodents using a Mann-Whitney U-test, implemented in R 2.9.2 as Wilcoxon rank sum test with continuity correction. Since this test examines if two distributions differ significantly from each other, all beaver samples were included in the test.

We showed that mitochondrial substitution rates of *Castor* differed significantly from those of the other rodents in the dataset. An increase of the observed rates towards the tips in a phylogeny (Fig. 4) is assumed to be a result of transient polymorphisms present in the more recent timescales of a phylogeny [33]. Although this hypothesis is controversial [54], in our BEAST phylogeny the estimated substitution rates also increase towards the tips. In order to explore this possible bias due to sampling on different taxonomic levels, we averaged the substitution rates along all branches leading to the common ancestor of all tested taxa (Table S5) and repeated the Mann-Whitney U-tests with those average rates (Table 1). In order to average the rates, for each of the three tests (*Castor* vs. mouse-related clade, *Castor* vs. rest of rodents, *Castor* vs. rest of glires, as indicated in Table 1) the arithmetic mean was calculated from the rates along the branches leading to the ancestral node connecting the tested taxa. The resulting p-values indicated an even more pronounced, highly significant difference between the lower rates of beavers and the higher rates of the other rodents in the dataset (Table 1). Further, we correlated the substitution rates of rodents with life history traits, such as body mass, lifespan and age at sexual maturity [21] using linear regressions in R. In order to avoid unbalanced data, we collapsed all sampled beavers into one data point for each regression. Measurements of substitution rates and life history traits for beavers were therefore averaged using the arithmetic mean. References for maximum lifespan, body mass and age at sexual maturity are given in Table S7 and Supporting References S1.

## Supporting Information

Table S1 Samples and overview of processing for mitochondrial genome sequencing. Samples were obtained from different source populations of beavers [S1], enriched for mitochondrial DNA and barcoded before sequencing. LR PCR: long range PCR. Hyb: hybridization capture.
(0.04 MB DOC)

Table S2 Taxa and accession numbers of the mitochondrial genome sequences used. Sequences marked by * are additional outgroup sequences to rodents and were used to evaluate the topology of our phylogenetic tree in the initial ML analysis but were excluded in the later ML, NJ, MP, and Bayesian analyses due to computational constraints.
(0.06 MB DOC)

Table S3 Bootstrap support for monophyletic clades in maximum likelihood analyses of rodent mitochondrial genome alignments of different length. Support values for clades recovered in previous studies decline when using smaller fractions of the sequence data. Support values are given for alignments including 24 taxa and 39 taxa separated by a slash. Note that all alignments excluded a less well aligned region containing the control region.
(0.04 MB DOC)

Table S4 Fossil calibrations and age estimates. Prior and posterior values for the time to the most recent common ancestor (tmrca) of monophyletic clades determined by BEAST analyses are given in million years ago (mya). L.: late. M.: middle. HPD: highest posterior density. Stderr: Standard error.
(0.05 MB DOC)

Table S5 Mitochondrial substitution rates for glires. Tip rates estimated by BEAST and averaged rates given in substitutions per position per million years.
(0.06 MB DOC)

Table S6 The relationships of body mass, life history traits and substitution rates were explored in linear regressions. A linear model correlated the estimated rates with lifespan and reached a significance level below 0.05 for tip as well as averaged rates. In contrast, linear models correlating rates with body mass and age at sexual maturity did not reach this significane level. Data on the body mass of the sequenced Anomalurus sp. individual were missing. Thus independent regressions were made for datasets containing Anomalurus with a body mass of 700 g and 2000 g. *: significant, <0.05.
(0.04 MB DOC)

Table S7 Life history traits of rodents and references. Since no data were available for M. kikuchii, data for M. oeconomus was used instead. Both taxa are closely related [S19]. Note that high values for lifespan could result from animals held in captivity.
(0.07 MB DOC)

Table S8 Oligo sequences. Long range PCR primer and blocking oligos.
(0.05 MB DOC)

Supporting References S1 Supporting References
(0.03 MB DOC)
