# Supplementary material for: Mitochondrial Genomes Reveal Slow Rates of Molecular Evolution and the Timing of Speciation in Beavers (Castor), One of the Largest Rodent Species
Source: PLoS One. 2011 Jan 28;6(1):e14622. doi: 10.1371/journal.pone.0014622 (PMC3030560; doi:10.1371/journal.pone.0014622)
Supplement: Table S1 — Samples and overview of processing for mitochondrial genome sequencing. Samples were obtained from different source populations of beavers [S1], enriched for mitochondrial DNA and barcoded before sequencing. LR PCR: long range PCR. Hyb: hybridization capture. (0.04 MB DOC) [file pone.0014622.s001.doc]

**Table S1.** **Samples and overview of processing for mitochondrial genome sequencing.**

| **Sample** | **Collection – ID** | **Source population** | **Enrichment** | **Barcoding protocol** |
| --- | --- | --- | --- | --- |
| *Castor canadensis* | Finnish Game and Fisheries Research Institute - Savitaipale 22.4.2006 | *C. canadensis* in Eastern Scandinavia (population introduced from North America) | LR PCR | [S2] |
| *Castor fiber* al | Zoologische Sammlungen Leipzig - M 85 / 2007 | *C. fiber* ssp. *albicus*, central Europe | LR PCR | [S2] |
| *Castor fiber* in | Helmholtz Centre - C.f. 86 | *C. fiber* ssp. *belorussicus* and *orientoeuropaeus*, eastern Europe and western Asia | LR PCR | [S2] |
| *Castor fiber* bi | Helmholtz Centre - C.f. 121 | *C. fiber* ssp. *birulai*, Asia | LR PCR | [S2] |
| *Castor fiber* tu | Helmholtz Centre - C.f. 161 | *C. fiber* ssp. *tuvinicus*, Asia | Hyb | [S3] |
| *Castor fiber* po | Helmholtz Centre - C.f. 134 | *C. fiber* ssp. *pohlei*, Asia | Hyb | [S3] |

Samples were obtained from different source populations of beavers [S1], enriched for mitochondrial DNA and barcoded before sequencing. LR PCR: long range PCR. Hyb: hybridization capture.
