# Supplementary material for: Mitochondrial Genomes Reveal Slow Rates of Molecular Evolution and the Timing of Speciation in Beavers (Castor), One of the Largest Rodent Species
Source: PLoS One. 2011 Jan 28;6(1):e14622. doi: 10.1371/journal.pone.0014622 (PMC3030560; doi:10.1371/journal.pone.0014622)
Supplement: Table S2 — Taxa and accession numbers of the mitochondrial genome sequences used. Sequences marked by * are additional outgroup sequences to rodents and were used to evaluate the topology of our phylogenetic tree in the initial ML analysis but were excluded in the later ML, NJ, MP, and Bayesian analyses due to computational constraints. (0.06 MB DOC) [file pone.0014622.s002.doc]

Table S2. Taxa and accession numbers of the mitochondrial genome sequences used.

| **Species** | **Accession number** |
| --- | --- |
| *Ochotona collaris* | AF348080.1 |
| *Oryctolagus cuniculus* | AJ001588.1 |
| *Lepus europaeus* | AJ421471.1 |
| *Cavia porcellus* | NC_000884 |
| *Jaculus jaculus* | NC_005314 |
| *Myoxus glis* | NC_001892 |
| *Thryonomys swinderianus* | NC_002658 |
| *Nannospalax ehrenbergi* | NC_005315 |
| *Sciurus vulgaris* | NC_002369 |
| *Anomalurus* sp. | NC_009056 |
| *Microtus kikuchi* | NC_003041 |
| *Cricetulus griseus* | NC_007936 |
| *Rattus norvegicus* | NC_001665 |
| *Mus musculus* | NC_006915 |
| *Cebus albifrons** | AJ309866.1 |
| *Hylobates lar** | X99256.1 |
| *Pongo pygmaeus** | D38115.1 |
| *Gorilla gorilla* | NC_001645.1 |
| *Homo sapiens* | J01415.2 |
| *Pan troglodytes** | D38113.1 |
| *Chlorocebus sabaeus** | EF597503.1 |
| *Papio hamadryas** | Y18001.1 |
| *Macaca sylvanus* | AJ309865.1 |
| *Colobus guereza** | AY863427.1 |
| *Trachypithecus obscurus** | AY863425.1 |
| *Cynocephalus variegates** | AJ428849.1 |
| *Nycticebus coucang** | AJ309867.1 |
| *Tarsius bancanus** | AF348159.1 |
| *Lemur catta* | AJ421451.1 |
| *Talpa europaea** | Y19192.1 |
| *Ovis aries** | AY858379.1 |
| *Canis familiaris** | CFU96639 |
| *Tupaia belangeri** | AJ421453.1 |

Sequences marked by * are additional outgroup sequences to rodents and were used to evaluate the topology of our phylogenetic tree in the initial ML analysis but were excluded in the later ML, NJ, MP, and Bayesian analyses due to computational constraints.
