# Supplementary material for: Mitochondrial Genomes Reveal Slow Rates of Molecular Evolution and the Timing of Speciation in Beavers (Castor), One of the Largest Rodent Species
Source: PLoS One. 2011 Jan 28;6(1):e14622. doi: 10.1371/journal.pone.0014622 (PMC3030560; doi:10.1371/journal.pone.0014622)
Supplement: Table S3 — Bootstrap support for monophyletic clades in maximum likelihood analyses of rodent mitochondrial genome alignments of different length. Support values for clades recovered in previous studies decline when using smaller fractions of the sequence data. Support values are given for alignments including 24 taxa and 39 taxa separated by a slash. Note that all alignments excluded a less well aligned region containing the control region. (0.04 MB DOC) [file pone.0014622.s003.doc]

**Table S3. Bootstrap support for monophyletic clades in maximum likelihood analyses of rodent mitochondrial genome alignments of different length.**

| **Bootstrap support for reciprocally monophyletic groups ( 24 taxa / 39 taxa)** | **16,352 bp, annotated and non-annotated loci** | **15,865 bp, all annotated loci** | **14,270 bp, annotated loci excluding tRNAs and the replication origin** |
| --- | --- | --- | --- |
| Lagomorpha | 100/100 | 100/100 | 99/100 |
| Rodents | 70/52 | 69/45 | 42 / - |
| Squirrel clade | 81/78 | 85/75 | 68/63 |
| Ctenohystrica | 100/100 | 100/100 | 100/100 |
| Mouse clade | 76/80 | 77/76 | 41/59 |

Support values for clades recovered in previous studies decline when using smaller fractions of the sequence data. Support values are given for alignments including 24 taxa and 39 taxa separated by a slash. Note that all alignments excluded a less well aligned region containing the control region.
