# Supplementary material for: Mitochondrial Genomes Reveal Slow Rates of Molecular Evolution and the Timing of Speciation in Beavers (Castor), One of the Largest Rodent Species
Source: PLoS One. 2011 Jan 28;6(1):e14622. doi: 10.1371/journal.pone.0014622 (PMC3030560; doi:10.1371/journal.pone.0014622)
Supplement: Table S4 — Fossil calibrations and age estimates. Prior and posterior values for the time to the most recent common ancestor (tmrca) of monophyletic clades determined by BEAST analyses are given in million years ago (mya). L.: late. M.: middle. HPD: highest posterior density. Stderr: Standard error. (0.05 MB DOC) [file pone.0014622.s004.doc]

**Table S4. Fossil calibrations and age estimates.**

| **Clade** | **Tmrca prior used for calibration (mean/stdev)** | **Fossil calibrations and References** | **Tmrca posterior, mean (95% HPD lower, 95% HPD upper, stderr of mean)** | **Tmrca posterior, median** |
| --- | --- | --- | --- | --- |
| Catarrhini (*Homo*, *Gorilla*, *Macaca*) | 25-35 (30/2) | Cercopithecidae like *Macaca* are known from the early Miocene to recent and Hominidae like *Gorilla* and *Homo* from the early Pliocene to recent. Fossil Propliopithecidae (L. Eocene through M. Oligocene, Fayûm quarries, Africa) could possibly be one of the earliest members of the Hominoidea [4]. | 29.71 (25.92, 33.51, 2.4284E-2) | 29.71 |
| *Lemur*, *Homo*, *Gorilla*, *Macaca* | 63-90 (78/6) | The primate fossil record includes around 180 species since the basal Eocene epoch (54–55 Myr ago) [5,6]. Lower preservation rates for mammals in the Cretaceous period than during the Cenozoic supported the view that primates originated towards the close of the Cretaceous [6] with the early Tertiary relatives Adapidae (‘lemuroids’) and Omomyidae (‘tarsioids’) [7] or based on a model of the diversification pattern, possibly already more than 80 mya [6,7]. | 72.40 (62.88, 82.23, 8.154E-2) | 72.33 |
| Glires (Lagomorphs, rodents) | 55.4-90 (74/7) | Rodents appeared with Paramyidae in the later Paleocene of North America and the earliest Eocene of Europe [8]. Early rodents were, e.g. *Tribosphenomys* from the Paleocene of Central Asia [9]. Late Paleocene *Heomys* and *Mimotona* from China were considered to be close to the ancestral stock of Rodentia and Lagomorpha, respectively [10,11]. Glires may share a common ancestry with the Late Cretaceous Zalambdalestidae (*Kulbeckia kulbecke*, from the 85-90 my old Dzharakuduk fauna, Uzbekistan [12]) | 70.20 (61.12, 79.59, 8.7444E-2) | 70.13 |
| Lagomorphs | 37-90 (64/11) | Eocene *Dawsonolagus antiquus* from China [13], early Eocene Lagomorpha from Western India [14]. Glires may share a common ancestry with the Late Cretaceous Zalambdalestidae [12]. | 56.21 (44.55, 67.43, 0.129) | 56.34 |
| Rodents | - |  | 66.46 (57.15, 75.46, 9.2357E-3) | 66.40 |
| Cricetidae, Muridae | 19-28 (24/2) | The Murid *Antemus* from Siwalik, Pakistan (10-14mya) [15]. *Antemus thailandicus* from the middle Miocene of northern Thailand [16,17]. Features of Cricetids appeared in the late Oligocene (*Eucricetodon* from Mongolia) [18]. | 25.54 (21.94, 28.91, 2.7813E-2) | 25.53 |
| Muridae (*Rattus*, *Mus*) | 7-15 (11/1.5) | The Murid *Antemus* from Siwalik, Pakistan (10-14mya) [15]. *Progonomys debruijni* from Siwalik, Pakistan (8mya) seen as closely related to ancestral *Mus*. *Karnimata* (found in associatin with *Progonomys*) more closely related to *Rattus*. | 12.26 (9.65, 14.65, 2.2262E-2) | 12.25 |
| *Castor, Anomalurus* | - |  | 53.89 (43.46, 63.85, 0.1235) | 53.97 |
| *Castor* | - |  | 7.97 (3.66, 12.99, 0.1211) | 7.64 |
| *C. fiber* | - |  | 0.21 (0.11, 0.34, 1.6832E-3) | 0.20 |

Prior and posterior values for the time to the most recent common ancestor (tmrca) of monophyletic clades determined by BEAST analyses are given in million years ago (mya). L.: late. M.: middle. HPD: highest posterior density. Stderr: Standard error.
