# Supplementary material for: Mitochondrial Genomes Reveal Slow Rates of Molecular Evolution and the Timing of Speciation in Beavers (Castor), One of the Largest Rodent Species
Source: PLoS One. 2011 Jan 28;6(1):e14622. doi: 10.1371/journal.pone.0014622 (PMC3030560; doi:10.1371/journal.pone.0014622)
Supplement: Table S5 — Mitochondrial substitution rates for glires. Tip rates estimated by BEAST and averaged rates given in substitutions per position per million years. (0.06 MB DOC) [file pone.0014622.s005.doc]

**Table S5. Mitochondrial substitution rates for glires.**

| **Taxon** | **Median rate for tip branches** | **Median rates averaged proportional to branchlengths from tips to common ancestor of** | | |
| --- | --- | --- | --- | --- |
|  |  | **Mouse related clade** | **Rodents** | **Glires** |
| *Ochotona collaris* | 0.0092 |  |  | 0.0091 |
| *Oryctolagus cuniculus* | 0.0108 |  |  | 0.0078 |
| *Lepus europaeus* | 0.0092 |  |  | 0.0074 |
| *Cavia porcellus* | 0.0129 |  | 0.0125 | 0.0124 |
| *Jaculus jaculus* | 0.015 | 0.0144 | 0.0144 | 0.0141 |
| *Myoxus glis* | 0.0107 |  | 0.0105 | 0.0105 |
| *Thryonomys swinderianus* | 0.0163 |  | 0.0146 | 0.0144 |
| *Nannospalax ehrenbergi* | 0.0158 | 0.0145 | 0.0145 | 0.0143 |
| *Sciurus vulgaris* | 0.0094 |  | 0.0094 | 0.0095 |
| *Anomalurus* sp. | 0.0148 | 0.0141 | 0.0141 | 0.0139 |
| *Microtus kikuchi* | 0.0204 | 0.0155 | 0.0155 | 0.0153 |
| *Cricetulus griseus* | 0.0243 | 0.0167 | 0.0167 | 0.0164 |
| *Rattus norvegicus* | 0.021 | 0.0147 | 0.0147 | 0.0144 |
| *Mus musculus* | 0.023 | 0.0151 | 0.0151 | 0.0148 |
| *Castor fiber* ssp. *pohlei* | 0.0105 | 0.0104 | 0.0104 | 0.0104 |
| *Castor fiber* ssp. *tuvinicus* | 0.0097 | 0.0104 | 0.0104 | 0.0104 |
| *Castor fiber* ssp. *birulai* | 0.0128 | 0.0104 | 0.0104 | 0.0104 |
| *Castor fiber* ssp. *belorussicus* and *orientoeuropaeus* | 0.012 | 0.0104 | 0.0104 | 0.0104 |
| *Castor fiber* ssp. *albicus* | 0.0106 | 0.0104 | 0.0104 | 0.0104 |
| *Castor canadensis* | 0.0105 | 0.0102 | 0.0102 | 0.0102 |

Tip rates estimated by BEAST and averaged rates given in substitutions per position per million years.
