# Supplementary material for: Mitochondrial Genomes Reveal Slow Rates of Molecular Evolution and the Timing of Speciation in Beavers (Castor), One of the Largest Rodent Species
Source: PLoS One. 2011 Jan 28;6(1):e14622. doi: 10.1371/journal.pone.0014622 (PMC3030560; doi:10.1371/journal.pone.0014622)
Supplement: Table S6 — The relationships of body mass, life history traits and substitution rates were explored in linear regressions. A linear model correlated the estimated rates with lifespan and reached a significance level below 0.05 for tip as well as averaged rates. In contrast, linear models correlating rates with body mass and age at sexual maturity did not reach this significane level. Data on the body mass of the sequenced Anomalurus sp. individual were missing. Thus independent regressions were made for datasets containing Anomalurus with a body mass of 700 g and 2000 g. *: significant, <0.05. (0.04 MB DOC) [file pone.0014622.s006.doc]

**Table S6. The relationships of body mass, life history traits and substitution rates were explored in linear regressions**.

|  | **Lifespan** | **Body mass (Anomalurus 700g)** | **Body mass (Anomalurus 2000g)** | **Age at sexual maturity** |
| --- | --- | --- | --- | --- |
| **Tip rates** |  |  |  |  |
| R squared | 0.3402 | 0.0776 | 0.0784 | 0.2415 |
| P-value | 0.0225 * | 0.3147 | 0.3122 | 0.0881 |
| **Averaged rates** |  |  |  |  |
| R squared | 0.4011 | 0.0651 | 0.0601 | 0.0857 |
| P-value | 0.0113 * | 0.3586 | 0.3783 | 0.3319 |

A linear model correlated the estimated rates with lifespan and reached a significance level below 0.05 for tip as well as averaged rates. In contrast, linear models correlating rates with body mass and age at sexual maturity did not reach this significane level. Data on the body mass of the sequenced *Anomalurus* sp. individual were missing. Thus independent regressions were made for datasets containing *Anomalurus* with a body mass of 700g and 2000g. *: significant, <0.05.
