# Supplementary material for: Mitochondrial Genomes Reveal Slow Rates of Molecular Evolution and the Timing of Speciation in Beavers (Castor), One of the Largest Rodent Species
Source: PLoS One. 2011 Jan 28;6(1):e14622. doi: 10.1371/journal.pone.0014622 (PMC3030560; doi:10.1371/journal.pone.0014622)
Supplement: Table S7 — Life history traits of rodents and references. Since no data were available for M. kikuchii, data for M. oeconomus was used instead. Both taxa are closely related [S19]. Note that high values for lifespan could result from animals held in captivity. (0.07 MB DOC) [file pone.0014622.s007.doc]

**Table S7. Life history traits of rodents and references.**

| **Species** | **Maximum lifespan (months)** | | **Body mass (g)** | | **Age at sexual maturity (days)** | |
| --- | --- | --- | --- | --- | --- | --- |
| *Ochotona collaris* | 72 | [S20] | 129 | [S20] | 365 | [S21] |
| *Oryctolagus cuniculus* | 216 | [S20] | 1590 | [S22] | 107 | [S22] |
| *Lepus europaeus* | 144 | [S20] | 3820 | [S20] | 180 | [S23] |
| *Cavia porcellus* | 177.6 | [S20] | 728 | [S20] | 28 | [S24] |
| *Jaculus jaculus* | 48 | [S25] | 104 | [S26] | 300 | [S25,S27] |
| *Myoxus glis* | 108 | [S20] | 125 | [S22] | 365 | [S22] |
| *Thryonomys swinderianus* | 51.6 | [S20] | 5000 | [S22] | 502 | [S22] |
| *Nannospalax ehrenbergi* | 180 | [S28] | 200 | [S28,S29] | n.a. |  |
| *Sciurus vulgaris* | 144 | [S20] | 300 | [S22] | 282 | [S22] |
| *Anomalurus* sp. | 18 | [S30] | 700->1700 | [S31,S32] | n.a. |  |
| *Microtus oeconomus* | 21.6 | [33] | 58 | [33] | 30 | [33] |
| *Cricetulus griseus* | 36 | [34] | 42 | [34] | 84 | [34] |
| *Rattus norvegicus* | 60 | [S20] | 283 | [S20] | 64.6 | [S35] |
| *Mus musculus* | 72 | [S20] | 20 | [S22] | 35 | [S22] |
| *Castor fiber* | 216 | [S36] | 18000 | [S37] | 730 | [S38] |
| *Castor canadensis* | 252 | [S39] | 18600 | [S40] | 730 | [S22] |

Since no data were available for *M. kikuchii*, data for *M. oeconomus* was used instead. Both taxa are closely related [S19]. Note that high values for lifespan could result from animals held in captivity.
