# Supplementary material for: Mitochondrial Genomes Reveal Slow Rates of Molecular Evolution and the Timing of Speciation in Beavers (Castor), One of the Largest Rodent Species
Source: PLoS One. 2011 Jan 28;6(1):e14622. doi: 10.1371/journal.pone.0014622 (PMC3030560; doi:10.1371/journal.pone.0014622)
Supplement: Table S8 — Oligo sequences. Long range PCR primer and blocking oligos. (0.05 MB DOC) [file pone.0014622.s008.doc]

**Table S8.** **Long range PCR primer and blocking oligos.**

| **Long range PCR primer** | **5’-3’ sequence** |
| --- | --- |
| cf_mt_genome_fr1_F | CCCACCATCAGCACCCAAAGCTGACATTCTC |
| cf_mt_genome_fr1_R | WMGGCGGGAGAAGTAGATTGAAGCCAG |
| cf_mt_genome_fr2_F | AAGCTATCGGGCCCATACCCCGAAAATGTTGG |
| cf_mt_genome_fr2_R | GACATTCCCCGAGCGGGTTGCTGGTTTC |
| 080828_mtG_1F_Cf | AGAAACAAAGTCCCCACATCAGCACCCAAAGC |
| 080828_mtG_1F_Cc | GCTGACATTCTCATAATTAAACTACCCCCTGC |
| 080828_mtG_1R_biber | ACTTTCTTAGGGCTTTGAAGGCTCTTGG |
| 080828_mtG_2F_biber | GCAACCAAATACTTCATAACACAAGCCACAGC |
| 080828_mtG_2R_Cf | GGGACATTYCCCGAGCGGGTTGCTGG |
| **PCR primer, gap filling** | **5’-3’ sequence** |
| Cc_7900_mtG_F | CTCACCCTATTCATCATCTTCCAAC |
| Cc_7900_mtG_R | TTCGTTCATTTTTTTTCTTCAAGGG |
| Cf_10069_mtG_F | CACCACAACCTTAATTACTCTGAAC |
| Cf_10069_mtG_R | CCGTATGTATTTGATATTGTGACCAG |
| **Blocking oligos** | **5’-3’ sequence** |
| Flx_SusiH_blocking_A | CCATCTCATCCCTGCGTGTCCCATCTGTTCCCTCCCTGTCTCAG |
| Flx_SusiH_blocking_B | CCTATCCCCTGTGTGCCTTGCCTATCCCCTGTTGCGTGTCTCAG |
| Flx_SusiH_blocking_B_rc | CTGAGACACGCAACAGGGGATAGGCAAGGCACACAGGGGATAGG |
| Flx_SusiH_blocking_A_rc | CTGAGACAGGGAGGGAACAGATGGGACACGCAGGGATGAGATGG |
