# Supplementary material for: Mitochondrial Genomes Reveal Slow Rates of Molecular Evolution and the Timing of Speciation in Beavers (Castor), One of the Largest Rodent Species
Source: PLoS One. 2011 Jan 28;6(1):e14622. doi: 10.1371/journal.pone.0014622 (PMC3030560; doi:10.1371/journal.pone.0014622)
Supplement: Supporting References S1 — Supporting References (0.03 MB DOC) [file pone.0014622.s009.doc]

**Supporting references**

1. Durka W, Babik W, Ducroz J-F, Heidecke D, Rosell F, et al. (2005) Mitochondrial phylogeography of the Eurasian beaver *Castor fiber* L. Molecular Ecology 14: 3843-3856.

2. Meyer M, Stenzel U, Hofreiter M (2008) Parallel tagged sequencing on the 454 platform. Nat Protocols 3: 267-278.

3. Stiller M, Knapp M, Stenzel U, Hofreiter M, Meyer M (2009) Direct multiplex sequencing (DMPS): A novel method for targeted high-throughput sequencing of ancient and highly degraded DNA. Genome Research 19: 1843-1848.

4. Shoshani J, Groves C, Simons E, Gunnell G (1996) Primate Phylogeny: Morphological vs Molecular Results. Molecular Phylogenetics and Evolution 5: 102-154.

5. Gingerich P, Uhen M (1994) Time of origin of primates. Journal of Human Evolution 27: 443-445.

6. Tavare S, Marshall C, Will O, Soligo C, Martin R (2002) Using the fossil record to estimate the age of the last common ancestor of extant primates. Nature 416: 726-729.

7. Martin RD (1993) Primate origins: plugging the gaps. Nature 363: 223-234.

8. Wood AE (1959) Eocene Radiation and Phylogeny of the Rodents. Evolution 13: 354-361.

9. Lopatin AV, Averianov AO (2004) The Earliest Rodents of the Genus *Tribosphenomys* from the Paleocene of Central Asia. Doklady Biological Sciences 397: 336-337.

10. Douzery EJP, Huchon D (2004) Rabbits, if anything, are likely Glires. Molecular Phylogenetics and Evolution 33: 922-935.

11. Li C-K, Wilson RW, Dawson MR, Krishtalka L (1987) The origin of rodents and lagomorphs. In: HH Genoways (ed), Current Mammalogy, Plenum Press, New York 1: 97–108.

12. Archibald JD, Averianov AO, Ekdale EG (2001) Late Cretaceous relatives of rabbits, rodents, and other extant eutherian mammals. Nature 414: 62-65.

13. Li C, Meng J, Wang Y (2007) Dawsonolagus antiquus, A Primitive Lagomorph from the Eocene Arshanto Formation, Nei Mongol, China. Bulletin of Carnegie Museum of Natural History: 97-110.

14. Rose KD, DeLeon VB, Missiaen P, Rana RS, Sahni A, et al. (2008) Early Eocene lagomorph (Mammalia) from Western India and the early diversification of Lagomorpha. Proceedings of the Royal Society B: Biological Sciences 275: 1203-1208.

15. Jacobs L, Pilbeam D (1980) Of mice and men: Fossil-based divergence dates and molecular "clocks". Journal of Human Evolution 9: 551-555.

16. Jaeger J, Tong H, Buffetaut E, Ingavat R (1985) The first fossil rodents from the Miocene of northern Thailand and their bearing on the problem of the origin of the Muridae. Revue de paléobiologie 4: 1-7.

17. Catzeflis FM, Sheldon FH, Ahlquist JE, Sibley CG (1987) DNA-DNA hybridization evidence of the rapid rate of muroid rodent DNA evolution. Mol Biol Evol 4: 242-253.

18. Lindsay EH (1978) Eucricetodon asiaticus (Matthew and Granger), an Oligocene Rodent (Cricetidae) from Mongolia. Journal of Paleontology 52: 590-595.

19. Mekada K, Harada M, Lin L, Koyasu K, Borodin P, et al. (2001) Pattern of X-Y chromosome pairing in the Taiwan vole, Microtus kikuchii. Genome 44: 27-31.

20. Welch J, Bininda-Emonds O, Bromham L (2008) Correlates of substitution rate variation in mammalian protein-coding sequences. BMC Evolutionary Biology 8: 53.

21. Morrison SF, Hik DS (2007) Demographic analysis of a declining pika Ochotona collaris population: linking survival to broad-scale climate patterns via spring snowmelt patterns. Journal of Animal Ecology 76: 899-907.

22. Wooton JT (1987) The effects of body mass, phylogeny, habitat, and trophic level on mammalian age at first reproduction. Evolution 41: 732 - 749.

23. Stott P, Wight N (2004) Female reproductive tract abnormalities in european hares (Lepus europaeus) in Australia. J Wildl Dis 40: 696-703.

24. Nuwanyakpa M, Lukefahr SD, Gudahl D, Ngoupayou JD (1997) The current stage and future prospects of guinea pig production under smallholder conditions in West Africa; 1. Global overview. Livestock Research for Rural Development 9.

25. Happold D (1967) Biology of jerboa, Jaculus jaculus butleri (Rodentia, Dipodidae), in the Sudan. Journal of Zoology 151: 257-274.

26. Morrow E, Fricke C (2004) Sexual selection and the risk of extinction in mammals. Proceedings of the Royal Society of London Series B: Biological Sciences 271: 2395-2401.

27. Ehrlich C (2007) Kleinsäuger auf dem Weg zum Heimtier - Springmäuse. Zoologischer Zentral Anzeiger.

28. Magalhaes JPd, Costa J, Church GM (2007) An Analysis of the Relationship Between Metabolism, Developmental Schedules, and Longevity Using Phylogenetic Independent Contrasts. The Journals of Gerontology Series A: Biological Sciences and Medical Sciences 62: 149-160.

29. Nowak RM (1999) Walker's Mammals of the World. Johns Hopkins University Press Baltimore 1.

30. Schunke AC (2005) Systematics and Biogeography of the African Scaly-tailed Squirrels (Mammalia: Rodentia: Anomaluridae). Dissertation Bonn, Germany.

31. Kuhn H-Jr (1966) Anomalurus pelii auzembergeri in Liberia. Journal of Mammalogy 47: 334-338.

32. Website (2010) http://animaldiversity.ummz.umich.edu/site/accounts/information/ Anomalurus_beecrofti.html.

33. Website (2010) http://genomics.senescence.info/species/entry.php?species=Microtus _oeconomus.

34. Website (2010) http://www.napak.com/chinese_hamster.html.

35. Clark BR, Price EO (1981) Sexual maturation and fecundity of wild and domestic Norway rats (Rattus norvegicus). J Reprod Fertil 63: 215-220.

36. Heidecke D (2005) Meister Bockert - erst gefördert, jetzt geduldet. Unsere Jagd 56.

37. Djoshkin WW, Safonow WG (1972) Die Biber der alten und neuen Welt. Die Neue Brehm-Bücherei, A Ziemsen Verlag, Germany.

38. Fritzsche R (1931) Über Biberzucht. Pelztierzucht 7: 165-167.

39. Busher PE, Jenkins SH (1979) *Castor canadensis*. Mammalian Species 20.

40. Baker BW, Hill EP (2003) Beaver (*Castor canadensis*). In: Feldhamer, G A, BC Thompson, and JA Chapman Wild Mammals of North America: biology, management, and conservation Baltimore, MD The Johns Hopkins University Press.
